# Screening Genetic Resources of *Capsicum* Peppers in Their Primary Center of Diversity in Bolivia and Peru

**DOI:** 10.1371/journal.pone.0134663

**Published:** 2015-09-24

**Authors:** Maarten van Zonneveld, Marleni Ramirez, David E. Williams, Michael Petz, Sven Meckelmann, Teresa Avila, Carlos Bejarano, Llermé Ríos, Karla Peña, Matthias Jäger, Dimary Libreros, Karen Amaya, Xavier Scheldeman

**Affiliations:** 1 Bioversity International, Costa Rica Office, Turrialba, Costa Rica; 2 Bioversity International, Regional Office for the Americas, Cali, Colombia; 3 Inter-American Institute for Cooperation on Agriculture (IICA), San José, Costa Rica; 4 University of Wuppertal, Faculty of Mathematics and Natural Sciences, Department of Food Chemistry, Wuppertal, Germany; 5 Centro de Investigaciones Fitoecogenéticas de Pairumani (CIFP), Cochabamba, Bolivia; 6 Fundación Promoción e Investigación de Productos Andinos (PROINPA), Oficina Regional Valle Sur, Sucre, Bolivia; 7 Instituto Nacional de Innovación Agraria (INIA), Lima, Peru; Virginia Tech, UNITED STATES

## Abstract

For most crops, like *Capsicum*, their diversity remains under-researched for traits of interest for food, nutrition and other purposes. A small investment in screening this diversity for a wide range of traits is likely to reveal many traditional varieties with distinguished values. One objective of this study was to demonstrate, with *Capsicum* as model crop, the application of indicators of phenotypic and geographic diversity as effective criteria for selecting promising genebank accessions for multiple uses from crop centers of diversity. A second objective was to evaluate the expression of biochemical and agromorphological properties of the selected *Capsicum* accessions in different conditions. Four steps were involved: **1)** Develop the necessary diversity by expanding genebank collections in Bolivia and Peru; **2)** Establish representative subsets of ~100 accessions for biochemical screening of *Capsicum* fruits; **3)** Select promising accessions for different uses after screening; and **4)** Examine how these promising accessions express biochemical and agromorphological properties when grown in different environmental conditions. The Peruvian *Capsicum* collection now contains 712 accessions encompassing all five domesticated species (*C*. *annuum*, *C*. *chinense*, *C*. *frutescens*, *C*. *baccatum*, and *C*. *pubescens)*. The collection in Bolivia now contains 487 accessions, representing all five domesticates plus four wild taxa (*C*. *baccatum* var. *baccatum*, *C*. *caballeroi*, *C*. *cardenasii*, and *C*. *eximium*). Following the biochemical screening, 44 Bolivian and 39 Peruvian accessions were selected as promising, representing wide variation in levels of antioxidant capacity, capsaicinoids, fat, flavonoids, polyphenols, quercetins, tocopherols, and color. In Peru, 23 promising accessions performed well in different environments, while each of the promising Bolivian accessions only performed well in a certain environment. Differences in *Capsicum* diversity and local contexts led to distinct outcomes in each country. In Peru, mild landraces with high values in health-related attributes were of interest to entrepreneurs. In Bolivia, wild *Capsicum* have high commercial demand.

## Introduction

Emerging markets for high-value food products and the growing sophistication of consumers in developed and developing countries are providing unprecedented opportunities for small-scale farmers to make a transition from traditional production of low-value commodities towards the production of differentiated crops and varieties with greater value [1]. Crop diversity, specifically traditional varieties from centers of crop diversity such as the Andean region, can be a basis for differentiated market products, as demonstrated in the case of native potatoes and varietal chocolates [2], [3]. For most crops, however, this diversity remains under-researched. A small investment in screening this diversity for a wide range of traits is thus likely to reveal many traditional varieties with distinguished values that are of interest to breeders, growers and consumers.

This paper describes the process of identifying, from two large collections of native *Capsicum* in the crop´s main center of diversity, a promising set of germplasm for multiple uses including the development of differentiated products. To be able to select from these rich collections within a specific time span and budget, we applied a series of indicators of phenotypic and geographic diversity as selection criteria: botanical classification and geographic origin, then biochemical properties of *Capsicum* fruits, and finally agromorphological and biochemical evaluation data from trials in different environments.

**Fig 1:**
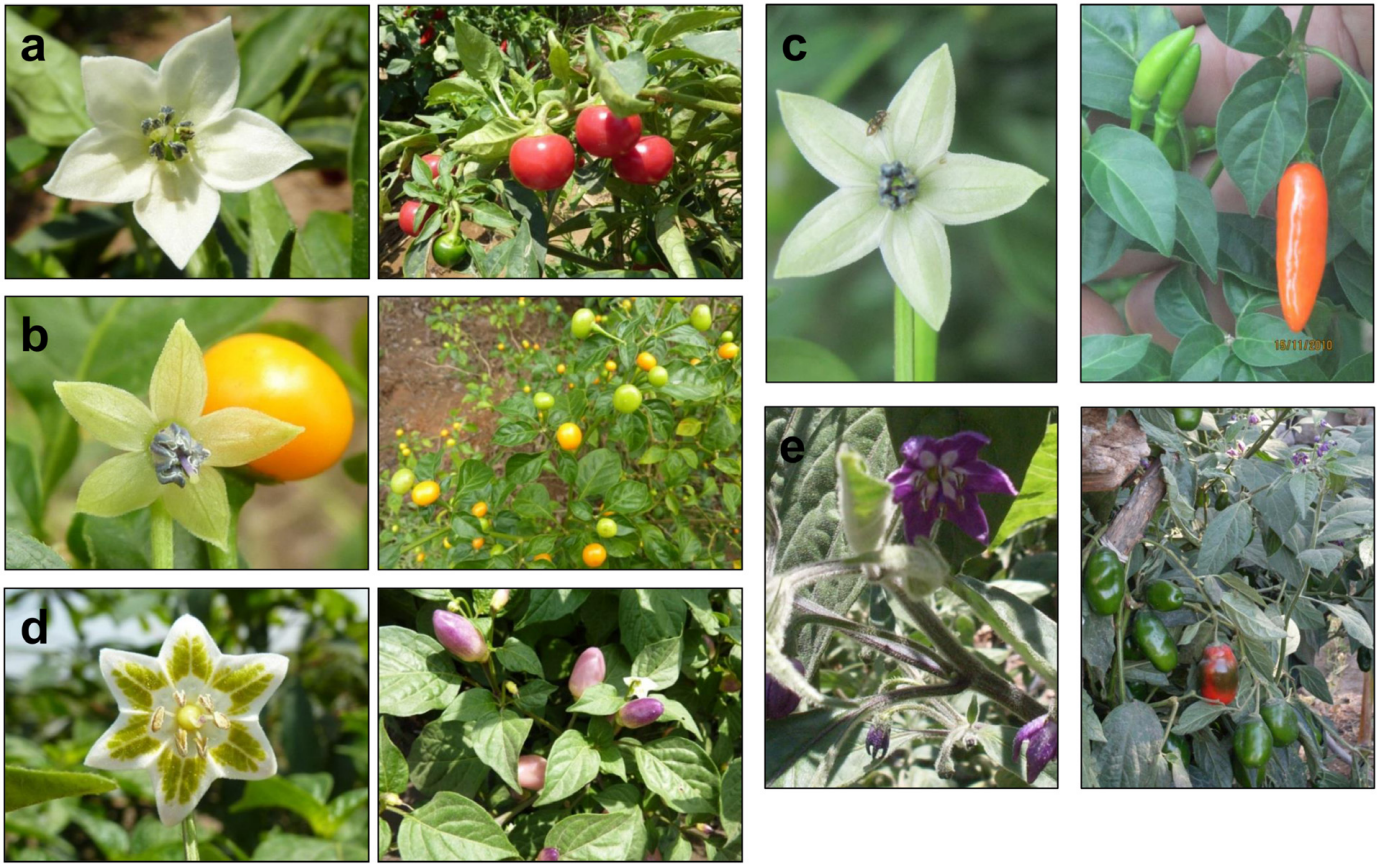
Examples of flowers and fruits from the five cultivated species: a) *C*. *annuum*; b) *C*. *chinense*; c) *C*. *frutescens*; d) *C*. *baccatum*; and e) *C*. *pubescens*. *Capsicum annuum* and her sister species, *C*. *chinense* and *C*. *frutescens* all have white flowers. The flowers of *Capsicum baccatum* have yellow-green spots on the white petals. *Capsicum pubescens* can be distinguished by purple flowers. Photo credits: Centro de Investigación y Desarrollo Rural Amazónico (CIDRA), Xavier Scheldeman, Maarten van Zonneveld.

We focused on *Capsicum* as a model crop because it is a highly diverse genus that offers abundant phenotypic variation. Although it is a well-known crop, its genetic diversity remains little studied. The genu*s* is endemic to the Americas, and for several millennia *Capsicum* has been an important dietary component throughout the New World [4]. Thirty-eight *Capsicum* species are currently recognized in USDA’s Genetic Resources Information Network (GRIN), of which five are domesticated: *C*. *annuum* L., *C*. *chinense* Jacq., *C*. *frutescens* L., *C*. *baccatum* L., and *C*. *pubescens* Ruiz & Pav. [5] (Fig 1).

**Fig 2:**
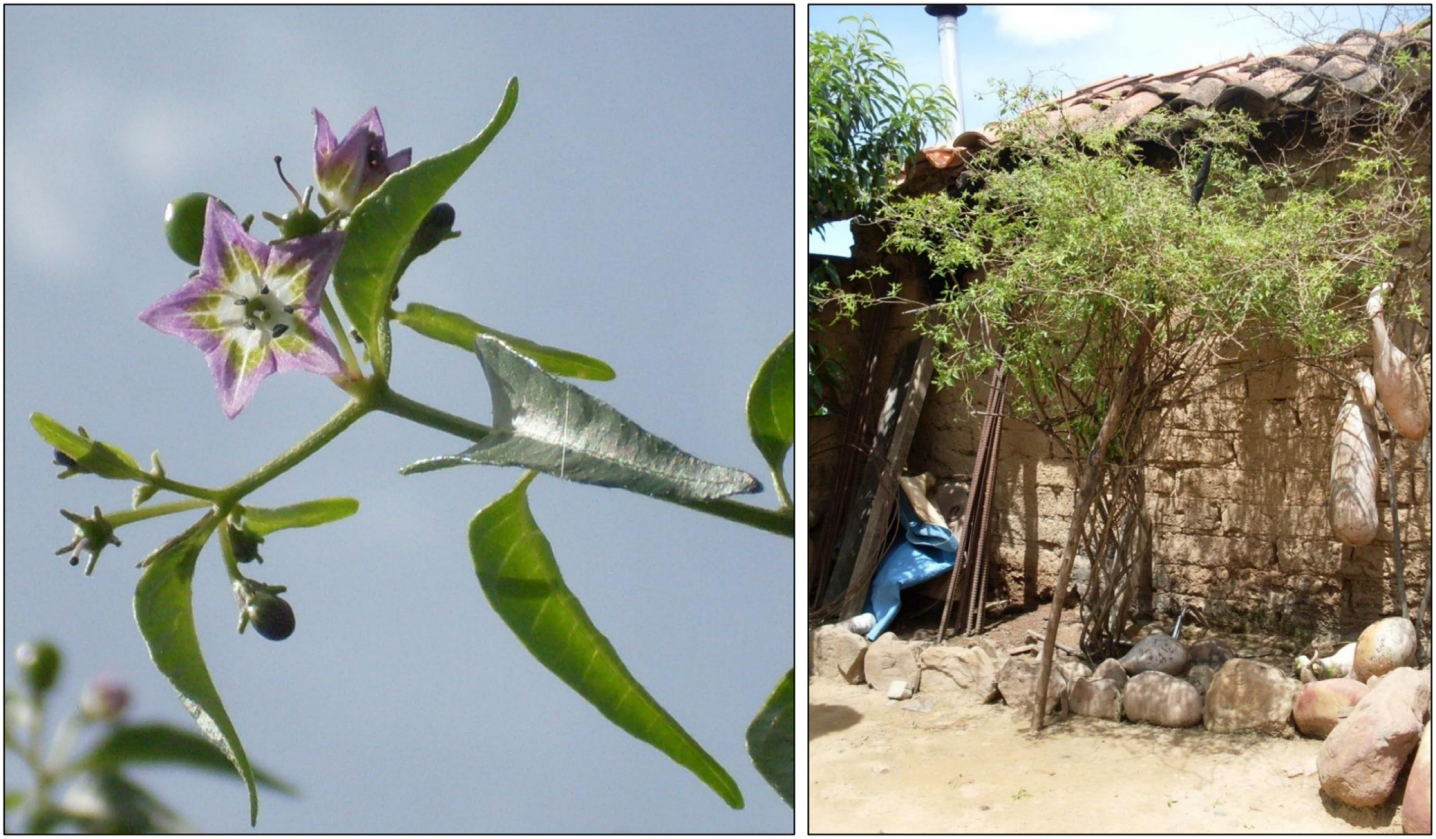
*Capsicum eximium* fruits, flowers and shrub in the backyard of a Bolivian farm, in Padilla, Chuquisaca. The fruits of this species are known as ´ulupica´. This is one of the several wild peppers, which is consumed in Bolivia. Photo credits: Maarten van Zonneveld.

Our research focused on Peru and Bolivia, which constitute the primary center of cultivated *Capsicum* diversity. Bolivia is one of America’s wild pepper hotspots [6], [7], where the fruits of several wild pepper species are consumed as food and used for medicinal purposes (Fig 2). Most wild peppers in other *Capsicum* hotspots, such as the Atlantic forests in Brazil, belong to an evolutionary distinct group, and are not consumed by humans [7], [8]. Peru is an important area of *Capsicum* diversification and is reportedly the country that harbors the greatest diversity of cultivated *Capsicum* in the world [9], [10]. Pepper species were cultivated in Peru as early as 4,000 years before present [11]. In pre-Columbian times, cultivated *Capsicum* species were probably transported to Peru from their centers of crop origin: *C*. *annuum* from Mexico; *C*. *chinense* from the Amazon basin, including its headwaters in Peru; while *C*. *baccatum* and *C*. *pubescens* were introduced from Bolivia, where they were probably first domesticated after the Neolithic revolution that started 12,000 years before present [6], [12], [13]. The fifth cultivated pepper, *C*. *frutescens* may have been domesticated in Central America or the Amazon basin [6].

After Columbus, cultivated *Capsicum* species, most notably *C*. *annuum*, quickly spread around the world, becoming a part of local cuisines and source of income to farmers worldwide [14]. *Capsicum* exports alone generated nearly US$ 5.7 billion in 2011, making it one of the most important vegetables and spices in international trade [15]. In several countries, native *Capsicum* diversity is gaining importance to develop processed food products for niche markets [16], [17]. *Capsicum* is also of interest for a wide range of other applications such as a natural colorant for food, an ingredient in pharmaceuticals, and a highly pungent extract used for self-defense sprays, animal repellents and insecticides [18].

Domesticated *Capsicum* species show wide morphological variability, including traits which are useful for discriminating among the species such as plant pubescence, number of flowers per node, calyx constriction, corolla color and spots, and seed color [19] (Fig 1), as well as variability in other, commercially valuable traits such as fruit color, shape, flavor, and biochemical components, including health and taste-related attributes like antioxidant capacity and capsaicinoids, the latter being responsible for *Capsicum*’s pungency [9], [20–22].

**Fig 3:**
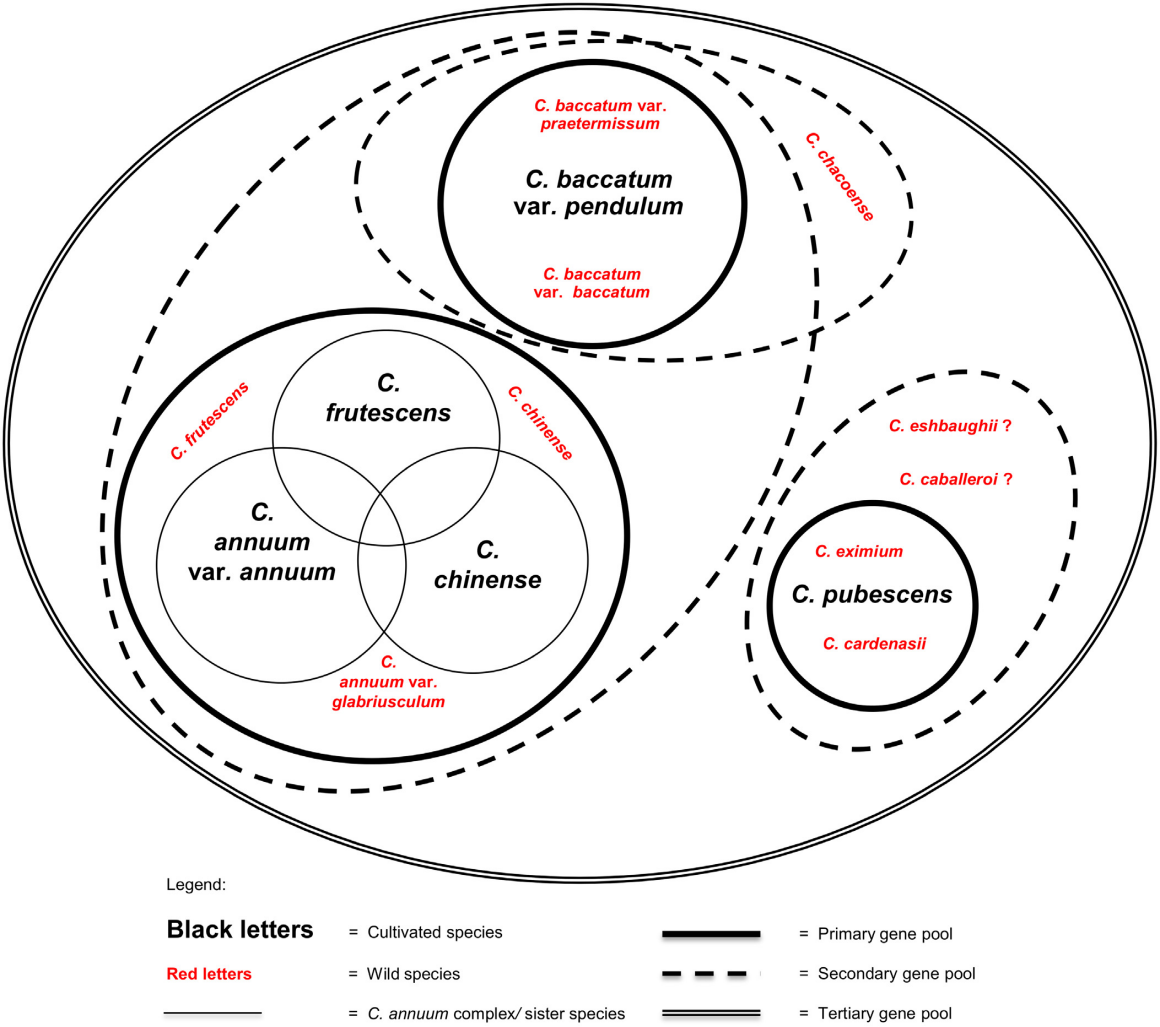
Cultivated *Capsicum* gene pools. *Capsicum annuum* L., *C*. *chinense* L. and *C*. *frutescens* L., while morphologically distinguishable, are largely interfertile with one another and are commonly regarded forming a species complex [24]. These three sister cultigens, together with their conspecific wild populations, including *C*. *annuum* var. *glabriusculum* (Dunal) Heiser & Pickersgill, constitute the *Capsicum annuum* primary gene pool. The *Capsicum baccatum* primary gene pool is formed by the cultivated *C*. *baccatum* L. or more specifically *C*. *baccatum* var. *pendulum* (Willd.) Eshbaugh; its conspecific wild relative *C*. *baccatum* var. *baccatum*; and the more distantly related species, *C*. *baccatum* var. *praetermissum* (Heiser & P. G. Sm.) Hunz. *Capsicum baccatum*’s secondary gene pool consists of *C*. *chacoense* Hunz. The *Capsicum pubescens* primary gene pool is made up of the cultivated species (*C*. *pubescens* Ruiz & Pav.), together with the closely related purple-flowered wild species *C*. *cardenasii* Heiser & P. G. Sm. and *C*. *eximium* Hunz. [24], [25]. Several other wild edible species, *C*. *eshbaughii* Barboza and *C*. *caballeroi* Nee are recently discovered in Bolivia and resemble *C*. *eximium* and *C*. *cardenasii* in certain fruit and flower aspects, but their relativeness to *C*. *pubescens* still needs to be clarified [42], [54]. And so there are other wild species that require further analysis. The little-studied wild species *Capsicum galapagoense* Hunz. and *C*. *tovarii* Eshbaugh et al. have been suggested by some authors to form part of the *C*. *annuum* complex and *C*. *baccatum* gene pools, respectively, but are intentionally left out of this diagram until more conclusive evidence supporting their inclusion becomes available. The cultivated *C*. *baccatum* var. *umbilicatum* (Vell.) Hunz. & Barboza is not included until further evidence confirms its distinction from *C*. *baccatum* var. *pendulum*.

The high degree of genetic diversity found within the genus provides both opportunities and challenges for plant breeders as the full extent and structure of this diversity remains underutilized and imperfectly understood. Crossing experiments and molecular marker studies have shown that the five domesticated species of *Capsicum* pertain to three distinct primary gene pools (*sensu* [23]), [24], [25], which are centered around *C*. *annuum*, *C*. *baccatum*, and *C*. *pubescens*, respectively, and are based on the degree of genetic proximity and reproductive compatibility with the target cultigen [26]. The three gene pools have little reproductive compatibility between them, although successful crosses have been reported between plants from the *C*. *annuum* and *C*. *baccatum* complexes [24], [27], [28]. Together, they include all of the cultivated landraces and local varieties pertaining to the five domesticates, as well as at least eight wild *Capsicum* taxa and conspecific wild populations (Fig 3).

Given the rich variety of *Capsicum* in its primary center of diversity, we expected that a wealth of commercially valuable properties for product development could be found in the cultivated and wild pepper species present in Peru and Bolivia, The specific objectives of this study were to: 1) demonstrate, with *Capsicum* as a model crop, the application of phenotypic and geographic indicators of diversity as effective criteria to select a set of promising accessions for multiple uses from crop centers of diversity; and 2) evaluate the expression of biochemical and agromorphological properties of the selected *Capsicum* accessions in different environmental conditions.

## Methods

**Fig 4:**
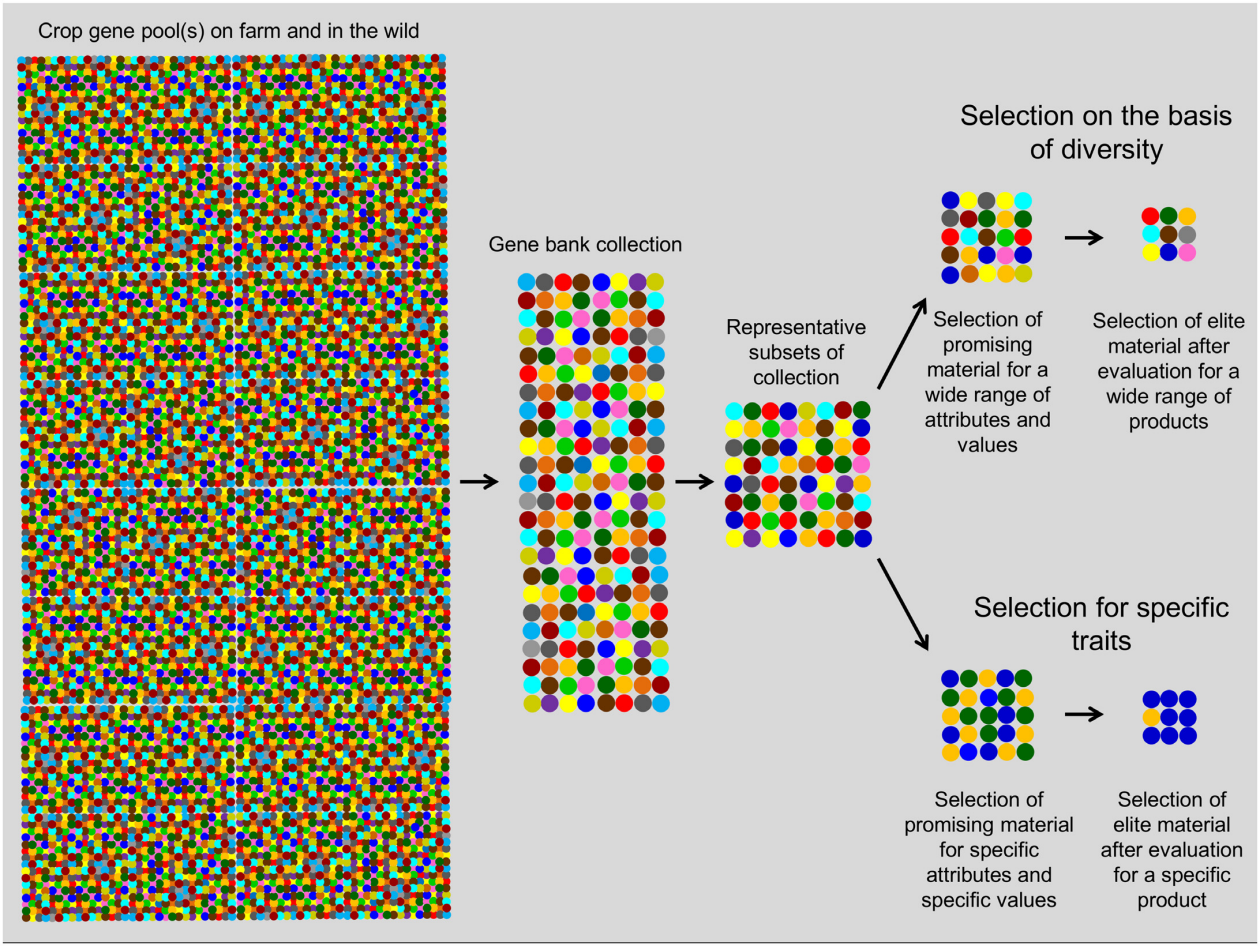
Conceptual diagram showing the different steps followed in this study in the conservation of the necessary variation for selection, and evaluation and identification of promising accessions on the basis of diversity indicators as selection criteria.

We followed four steps in the selection of promising accessions (Fig 4): 1) Strengthening and expansion of existing genebank collections; 2) biochemical characterization of *Capsicum* fruits from representative genebank subsets; 3) selection of promising accessions for multiple uses from the subsets; and 4) evaluation of promising accessions in different environments. All activities were conducted between 2010 and 2013 in Peru and Bolivia.

### 1) Strengthening and Expansion of Existing Genebank Collections

At the outset of this study, the Instituto Nacional de Innovación Agraria (INIA) from Peru maintained a collection of 106 accessions from only three regions of this country. During the study, INIA carried out eight collecting trips which combined the following criteria for determining the collection routes in Peru: 1) available knowledge on *Capsicum* diversity in Peru [17]; 2) geographic origin of existing genebank and herbarium accessions (see www.gbif.org); and 3) knowledge of INIA experts on *Capsicum* production areas.

The pre-existing Bolivian germplasm collection of 396 accessions held at the Centro de Investigaciones Fitoecogenéticas de Pairumani (CIFP) in Bolivia already included a wide range of native *Capsicum* diversity. Complementary collecting activities in search of landraces and wild *Capsicum* species in cloud forests and seasonally dry woodlands in the departments of La Paz, Cochabamba, Sucre and Chuquisaca were carried out.

Taxonomic identification of the Peruvian accessions was carried out by David E. Williams, while accessions of the Bolivian collection were botanically identified by Gloria Barbosa, Margoth Atahuachi and Ximena Reyes. Both collections were regenerated as part of this study following exclusion protocols to avoid cross pollination and to assure genetic integrity of the accessions. INIA and CIFP did not require specific permission for the collection locations as they were national authorities in conservation of plant genetic resources at the time of field collection.

### 2) Biochemical Characterization of *Capsicum* Fruits from Representative Genebank Subsets

Subsets of about 100 accessions per country, representing the overall diversity of each collection, were selected for biochemical characterization of *Capsicum* fruits (see Fig 4). Diversity was maximized by using available data on botanical classification and passport data of geographic origins as a proxy for genetic variation [29]. Fruit sampling was done during the seed regeneration of the accessions. Early-fruiting accessions had greater representation in the subsets compared to late-fruiting accessions because we only selected from accessions with mature fruits at the time of fruit collection.

Twelve seedlings were planted per accession, and of these up to six healthy plants were selected randomly for collection of bulk samples of fruits to be used in a first biochemical screening. In Peru, the accessions were all grown in a common plot in Huaral, Lima (Table 1). In Bolivia, the accessions were grown in five separate environments recognizing that some materials were highly adapted to specific climate zones, particularly the wild and semi-domesticated peppers like ‘arivivi’ (*C*. *baccatum* var. *baccatum*) and ‘ulupica’ (*C*. *eximium*) (Table 1). Fruit samples were shipped to Germany, as dried and crushed material after complying with Peruvian, Bolivian and German legal and phytosanitary regulations, for biochemical analysis.

**Table 1:** Passport data of characterization and evaluation sites in Bolivia and Peru.

Site	Climate zone according to Köppen^c^	Longitude (dd)^d^	Latitude (dd)^d^	Altitude (m)^e^	Annual mean temperature (C°)^e^	Annual precipitation (mm)^e^
Peru						
Chiclayo, Lambayeque	Hot desert	-79.78	-6.72	42	22.4	25
Pucallpa, Ucayali	Equatorial monsoon	-74.55	-8.38	153	26.4	1,667
Huaral, Lima	Hot desert	-77.21	-11.5	153	19.5	19
Tambo Grande, Piura	Hot desert	-80.94	-4.91	19	23.3	48
Bolivia						
Cochabamba, Cochabamba^a^ ^b^	Dry cold steppe	-66.16	-17.39	2,600	17.0	516
Mairana, Santa Cruz^a^	Dry hot steppe	-63.96	-18.12	1,349	20.7	653
Monteagudo, Chuquisaca^a^ ^b^	Warm temperature climate, dry winter and warm summer	-63.98	-19.82	1,156	21.6	864
Padilla, Chuquisaca^a^ ^b^	Warm temperature climate, dry winter and hot summer	-64.30	-19.30	2,129	18.2	657
Santa Cruz, Santa Cruz^a^ ^b^	Equatorial monsoon	-63.17	-17.80	428	24.0	1,244

^a^ Bolivian sites where Bolivian materials from the representative subset were grown for biochemical characterization.

^b^ Bolivian sites where promising Bolivian materials were grown for the genotype by environment (G x E) evaluation.

^c^ Köppen climate zone defined following methods explained by Cadima et al. (2014) [57].

^d^ Sites are georeferenced with GeoNames (www.geonames.org).

^e^ Climate data is derived from the downscaled 2.5 minutes resolution Worldclim dataset (http://www.worldclim.org).

Biochemical analyses followed Meckelmann *et al*. [9], [30] and included screening of antioxidant capacity (TEAC), levels of ASTA extractable color, capsaicinoids (responsible for pungency), fat, flavonoids, polyphenols, quercetin, and tocopherols (vitamin E) (Table 2). These phytonutrients influence taste, and/or are beneficial for nutrition and health [30], [31].

**Table 2:** Variables and methods of measurement for biochemical characterization of *Capsicum* samples.

Attributes	Method
Capsaicinoids (nordihydrocapsaicin, capsaicin, dihydrocapsaicin)	High-performance liquid chromatography (HPLC) with fluorescence detection
Flavonoids	HPLC analysis of quercetin, luteolin kaempferol and apigenin
Total polyphenols	Folin-Ciocalteu assay
Antioxidant capacity (TEAC)	ABTS assay
Vitamin E (α-, β- and γ-tocopherols)	HPLC with fluorescence detection
Extractable color (ASTA)	Photometric measurement of carotenoids after extraction with acetone
Fat	Near Infrared (NIR) and gravimetric micro method

For further details about the methods refer to Meckelmann et al. [9], [30].

### 3) Selecting Sets of Promising Accessions for Multiple Uses

About 40 promising accessions were selected in each country from the subsets based on the biochemical characterization of *Capsicum* fruits designed to capture a wide range of options for product development. Variation in pungency, differences in color, and accessions with high values for the other biochemical attributes were important selection criteria. We also included as many *Capsicum* species as possible since phenotypic differences may occur both within and between species.

### 4) Evaluation of Promising Accessions in Different Environments

Once promising accessions had been identified, they were grown in four evaluation sites each in Bolivia and Peru in different climate zones (Table 1). At each site, local management practices were followed (For Peru see S1 Table; for Bolivia see S2 Table). Three repetitions were established within each site. For each accession, 12 seedlings from its regenerated seed material were planted in a row with a distance of 70 cm between individuals. The distance between rows of accessions was 80 cm. The two outer seedlings of each accession were not considered in further studies to avoid border effects. A row of seedlings was planted as a buffer alongside the outer accessions to avoid border effects for these materials. Seedlings that died within two weeks were replaced.

In the evaluation sites, the promising accessions were sampled for a second round of biochemical analyses in Germany following the same methodology as explained above (i.e. fruit bulk samples from up to six healthy plants selected at random). These accessions were also morphologically characterized for a number of highly discriminating traits of agronomic and commercial interest according to the *Capsicum* descriptors developed by IPGRI *et al*. (1995) [19]. These traits were mean plant height (cm) per accession, flowering time (number of days to 50% plants flowering), mean fruit length (cm), weight (g) and width (cm).

We applied a two-way ANOVA to examine differences in biochemical and morphological attributes between species and locations. We identified these two factors as fixed following Annicchiarico (2002) [32], considering species as a group factor of promising accessions and locations as specific combinations of management and environment representing certain production areas. In case of differences, post-hoc Tukey`s Honest Significant Difference (HSD) analyses allowed significantly different groups to be identified.

Since data for several traits did not follow a normal distribution and in order to process data of all traits similarly, values of each trait were transformed into ranked data. These transformed data were then used in *F* analyses, *t* tests, HSD and multivariate analyses (see [33]). To reduce the false positive rate in the multiple trait tests, we adjusted the *p* values obtained with a False Discovery Rate (FDR) correction.

To facilitate further use of germplasm for different purposes, we classified promising accessions with similar trait-value combinations in separate groups. Trait values of each accession across locations were averaged and then ranked as explained above. We applied hierarchical clustering, calculating Euclidian distances between accessions with the rank scores and applying Ward´s method to group them. The final number of clusters was defined with the Kelley-Gardner-Sutcliffe penalty function [34]. A Principal Component Analysis (PCA) was done with these rank scores to visualize how traits related to each other, between species and among cluster groups.

We examined the phenotypic stability of the promising accessions for each trait by calculating environmental variance following Annicchiarico (2002) [32]:

$$S^{2}=\Sigma \frac{{\left(R_{ij}-\;m_{i}\right)}^{2}}{\left(e-1\right)}$$

In which *S* ^2^ is the environmental variance for a specific accession; *R*_*ij*_ is the average value for a particular trait in a certain location; *m*_*i*_ is the average value of an accession for a particular trait across locations; and *e* is the number of locations. We compared differences in phenotypic stability between the accessions with an ANOVA, in which the environmental variances measured per trait were considered as a repeated measure. To compare the environmental variances obtained for the different traits, we standardized the *S* ^2^ values for each trait between 0 and 1.

The environmental variance for an accession is usually large when its *m*_*i*_ value is high [35]. To identify traits for which accessions with high values are stable across environments, we correlated for each trait, the *m*_*i*_ values with the corresponding *S* ^2^ values.

Finally, we examined how consistent the plants of promising materials were in the expression of biochemical properties compared to the mother plants that were used for seed regeneration of each accession. Therefore, we compared for each trait, the *m*_*i*_ values from the genotype by environment (G *x* E) analysis with the results from the first biochemical screening. Pairwise *t* tests were applied to compare the rank scores.

Most analyses were carried out with basic statistical functions in the R statistical environment [36]; HSD analyses were done with the Agricolae package [37]; the PCA with help of the Vegan package [38]; the Kelley-Gardner-Sutcliffe penalty function was applied with the Maptree package [39].

As this work progressed and results of the germplasm screening became available, we shared the results with several actors in the *Capsicum* value chain: entrepreneurs, farmers and others convened at three meetings in Peru and two meetings in Bolivia in 2011 and 2012 [40], [41].

## Results

### 1) Strengthening and Expansion of Existing Genebank Collections

**Fig 5:**
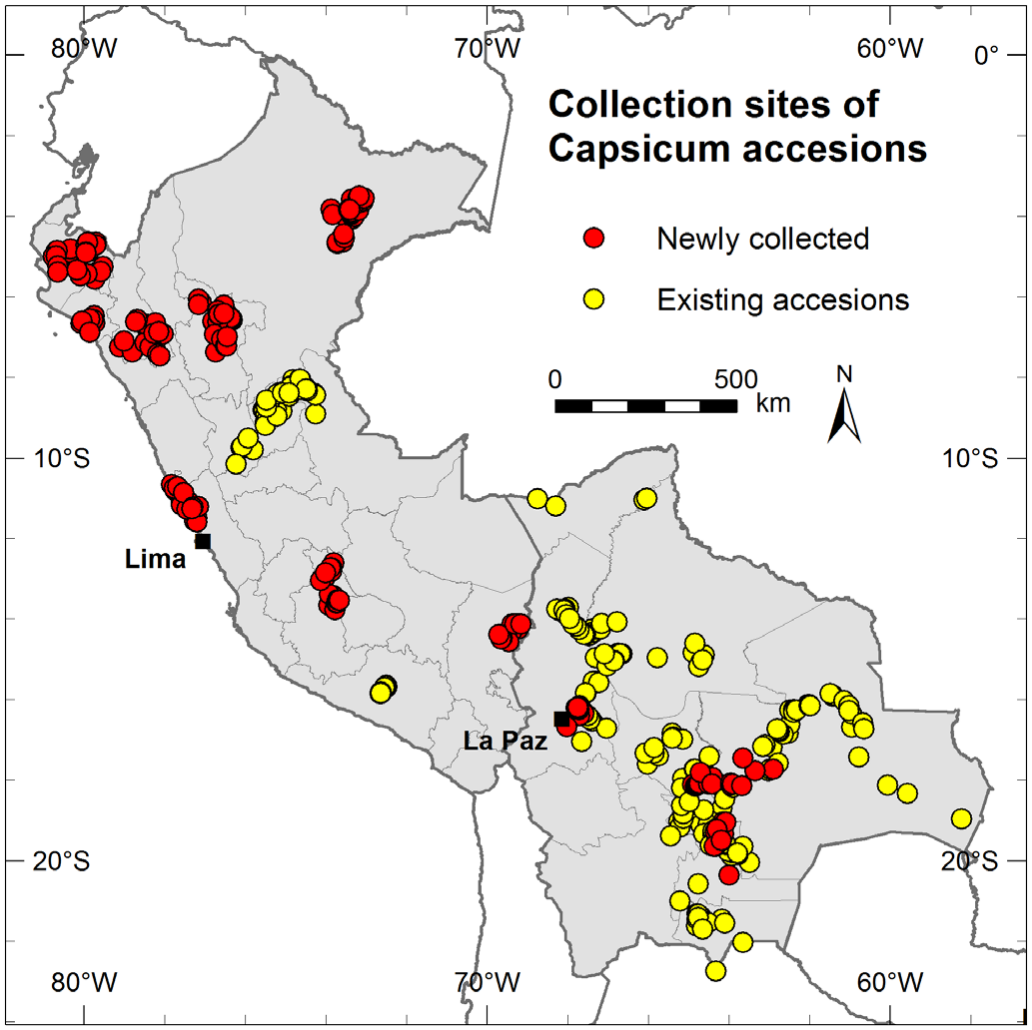
The Peruvian and Bolivian collection sites. Yellow dots refer to collection sites from the pre-existing accessions. Red dots refer to the sites where INIA (Peru) and CIFP (Bolivia) made complementary collections as part of this study.

In Peru, we established one of the most diverse national collections of native *Capsicum* varieties in the world consisting of 712 accessions encompassing the five domesticated species and dozens of locally recognized landraces [31] (Fig 5; Table 3), and a representative subset of 90 accessions from which 39 promising accessions were selected for evaluation in different environments.

**Table 3:** Taxonomic identification of the CIFP and INIA *Capsicum* collections.

Taxon	Accessions in CIFP collection (Bolivia)	Accessions in INIA collection (Peru)
Domesticated species		
*C*. *annuum* L.	12	46
*C*. *chinense* Jacq.	68	213
*C*. *frutescens* L.	25	48
*C*. *baccatum* L.	217	70
*C*. *pubescens* Ruiz & Pav.	13	301
Wild species		
*C*. *baccatum* var. *baccatum*	11	
*C*. *caballeroi* Nee	1	
*C*. *cardenasii* Heiser & P. G. Sm.	2	
*C*. *eximium* Hunz.	18	
Unidentified *Capsicum* accessions	120	34
Total	487	712

The CIFP collection in Bolivia now contains 487 accessions encompassing the five domesticated species and at least four wild taxa consumed by humans, including the recently discovered wild species *C*. *caballeroi* [42] (Fig 5; Table 3), which is unique to this collection. This collection conserves an important amount of *C*. *baccatum* variability compared to international *Capsicum* collections because Bolivia is a primary center of diversity of cultivated *C*. *baccatum* var. *pendulum* [6], [43] (S3 Table). Over 80% of the accessions are duplicated in the national Bolivian collection held by Instituto Nacional de Innovación Agropecuaria y Forestal (INIAF). A representative subset of 96 accessions was established for Bolivia from which 44 promising materials were selected.

### 2) Biochemical Characterization of *Capsicum* Fruits from Representative Genebank Subsets

Table 4 presents the mean values and ranges of biochemical attributes of the representative subsets in each country. For individual passport and biochemical characterization data please refer to S1 Dataset.

**Table 4:** Biochemical fruit screening of the *Capsicum* accessions from the representative subsets. Mean, minimum and maximum values are presented per species. In Peru, all accessions were grown in a common plot. In Bolivia, we anticipated that accessions were highly adapted to specific environments and were therefore grown in different locations.

	Antioxidant capacity (mmol/100g)	ASTA extractable color	Capsaicinoids (mg/100g)	Fat (g/100g)	Flavonoids (mg/100g)	Polyphenols (g/100g)	Quercetin (mg/100g)
Peru, Lima, Huaral							
*C*. *annuum* (n = 19)	4.2 (3.1–6.5)	64 (5–107)	312.0 (0.6–809.0)	9.1 (2.6–16.6)	9.4 (0.9–29.5)	1.77 (1.59–2.07)	8.0 (0.9–25.1)
*C*. *baccatum* (n = 26)	3.9 (2.8–5.4)	27 (1–66)	219.0 (52.1–711.7)	7.0 (2.2–17.1)	10.7 (0.7–27.4)	1.78 (1.44–2.59)	9.2 (0.7–25.1)
*C*. *chinense* (n = 43)	4.1 (2.1–9.2)	37 (4–146)	391.8 (1.2–1244.3)	7.8 (2.6–17.1)	4.5 (1.0–14.1)	1.83 (1.34–3.69)	4.2 (1.0–10.3)
*C*. *frutescens* (n = 2)	5.2 (3.4–7.0)	32 (3–60)	789.8 (404.1–1175.4)	9.1 (6.2–11.9)	2.3 (1.2–3.4)	2.08 (2.07–2.08)	2.3 (1.2–3.4)
Bolivia, Cochabamba, Cochabamba							
*C*. *annuum* (n = 1)	3.6	77	NA	6.7	4.0	1.53	3.2
*C*. *baccatum* (n = 10)	4.9 (4.0–5.7)	39 (5–101)	124.2 (11.9–313.7)	14.5 (7.6–20.4)	11.4 (2.7–29.9)	1.83 (1.34–2.13)	9.3 (2.0–24.3)
*C*. *pubescens* (n = 2)	4.4 (4.2–4.5)	15 (14–16)	230.4 (223–237.8)	7.1 (6.9–7.3)	2.6 (2.3–2.9)	1.89 (1.86–1.92)	2.2 (2.1–2.3)
Bolivia, Chuquisaca, Padilla							
*C*. *annuum* (n = 1)	4.2	13	NA	14.4	10.6	1.69	8.7
* *C*. *baccatum* var. *baccatum* (n = 1)	3.6	3	438.8	11.8	1.6	1.51	1.6
*C*. *baccatum* (n = 30)	4.1 (3.5–5.8)	46 (7–127)	30.0 (1.3–104.3)	11.8 (8.3–17.1)	11.3 (6.4–46.8)	1.74 (1.48–2.05)	9.5 (4.2–42.6)
* *C*. *eximium* (n = 3)	4.2 (3.9–4.4)	36 (11–61)	392.0 (304.1–454.5)	19.9 (18.9–21.4)	3.9 (0.4–6.6)	1.93 (1.69–2.19)	3.2 (0.4–6.0)
*C*. *frutescens* (n = 2)	3.7 (3.5–3.9)	24 (12–33)	64.6 (55.8–73.4)	15.4 (14.8–15.9)	10.8 (10.5–11.0)	1.56 (1.54–1.57)	8.8 (7.9–9.7)
Bolivia, Santa Cruz, Mairana							
* *C*. *baccatum* var. *baccatum* (n = 3)	4.2 (3.7–5.2)	30 (23–36)	315.4 (257.9–407.2)	23.7 (21.8–26.1)	8.4 (7.4–9.8)	1.45 (1.32–1.63)	6.6 (6.0–7.8)
*C*. *baccatum* (n = 16)	3.9 (3.3–4.9)	44 (11–84)	194.8 (32.6–415.8)	18.8 (12.0–32.8)	7.7 (3.7–13.8)	1.45 (1.21–1.74)	5.9 (2.2–11.0)
*C*. *frutescens* (n = 1)	5.0	29	781.3	14.8	4.2	1.86	2.8
Bolivia, Santa Cruz, Santa Cruz							
*C*. *annuum* (n = 1)	3.0	58	11.7	9.7	5.9	1.37	4.5
* *C*. *baccatum* var. *baccatum* (n = 2)	3.8 (3.6–3.9)	19 (15–23)	183.5 (32.8–334.1)	17.6 (15.7–19.4)	8.2 (4.4–12)	1.37 (1.31–1.43)	5.3 (2.9–7.7)
*C*. *baccatum* (n = 15)	3.8 (3.0–5.4)	50 (6–95)	145.6 (0.7–338.0)	14.0 (9.3–27.7)	4.5 (1.6–8.8)	1.40 (1.18–1.86)	3.1 (0.8–5.9)
*C*. *chinense* (n = 7)	4.0 (3.1–5.5)	59 (8–102)	174.9 (0.3–311.5)	11.7 (7.3–14.2)	3.8 (2.4–4.8)	1.41 (1.09–1.74)	2.7 (1.6–3.3)
*C*. *frutescens* (n = 1)	6.2	69	1027.9	17.1	4.5	2.05	3.7

* Wild taxa

The 90 accessions of the Peruvian subset included four of the five domesticated species (*C*. *annuum*, *C*. *baccatum*, *C*. *chinense and C*. *frutescens*) originating from ten regions. The biochemical results from Peruvian *C*. *pubescens* accessions are reported separately because they have a longer growing season compared to the other domesticates and were characterized separately [44].

In Bolivia, the subset of 96 accessions included the five domesticated species and two wild taxa, *C*. *eximium* and *C*. *baccatum* var. *baccatum*, from seven departments. The two wild taxa were of special interest to Bolivian entrepreneurs because of their potential for high-value product differentiation [41]. Accessions from other Bolivian wild pepper species like *C*. *caballeroi*, did not produce fruits in time for the biochemical screening and further selection.

### 3) Selecting sets of Promising Accessions for Multiple Uses

The 44 promising accessions from Bolivia included all species from the subset and covered a range from low to high pungency. Sixteen had exceptionally high values in one or more of the attributes of ASTA extractable color, antioxidant capacity, polyphenols, and fat content compared to the other accessions of the Bolivian subset. We did not find any differences in biochemical properties between the representative subset and the promising accessions according to posterior *t* tests (S4 Table).

In Peru, at the time of selecting promising materials, results of the biochemical analyses were only available for 24 of the subset accessions. From these, we selected eight promising materials which had high or low values in capsaicinoids and ASTA extractable color and/or high levels in antioxidant capacity, polyphenols or fat compared to the other Peruvian accessions that had been analyzed at that moment. Other promising accessions were selected later during a field visit to the regeneration site when most accessions were fruiting, with a special emphasis on variation in fruit form and color, to complete a total of 39 promising accessions representing the four species from the subset (see S1 Dataset). The variation in biochemical traits in the set of promising accessions did not differ compared to the representative subset (S4 Table).

### 4) Evaluation of Promising Accessions in Different Environments

In Bolivia, no promising accession produced fruits in time in two or more sites for the second round of biochemical analyses. Therefore, here we report only on the results of the Peruvian G *x* E evaluation experiment.

In Peru, fruits from 23 of the 39 accessions, in three of the four evaluation sites, were mature at the time planned for the second biochemical screening. In the fourth Peruvian site, Huaral, the accessions did not produce fruits in time for this round of biochemical analyses. Of the two promising *C*. *frutescens* accessions, only one produced fruits in the three sites. On its own it would not constitute a representative group, so we analyzed it together with the accessions of the genetically close *C*. *chinense* species (see [12], [25]).

For most accessions, sufficient fruits were only available for one single bulked sample per site for biochemical screening, while agromorphological evaluation was completed in all three repetitions in each site (S2 Dataset). Differences between repetitions were found for plant height between and within sites and for flowering within but not between sites (S5 Table). For one high-yielding accession, results of three repetitions in all sites were compared to confirm that biochemical stability within sites was higher than between sites [30]. To be able to compare the agromorphological performance of the 23 accessions with the biochemical expressions in their fruits, we averaged within each site agromorphological trait values from the three repetitions (S3 Dataset).

Eventually the species and location factors of our two-way ANOVA consisted of three germplasm groups and three evaluation sites respectively. The ANOVA revealed highly significant differences in performance between species for several traits and to a lower degree between different locations (Table 5). We observed most of these differences for the biochemical traits rather than the agromorphological features (Table 5).

**Table 5:** Analysis of variance (ANOVA) per trait and significance levels for the effects ‘species’ and ‘location’ and their interaction expressed as mean squares.

Attributes	Species	Location	S x L	Residuals
Antioxidant capacity	726.8 ^NS^	393.9 ^NS^	21.8 ^NS^	416.9
ASTA extractable color	3,843.0 ***	1,353.0 *	64.0 ^NS^	278.0
Capsaicinoids	670.3 ^NS^	558.8 ^NS^	18.1 ^NS^	414.0
Fat	5,507.0 ***	424.0 ^NS^	30 ^NS^	256
Flavonoids	2,451.9 ***	2,625.3 ***	156.4 ^NS^	276.2
Polyphenols	533.0 ^NS^	2,788.5 ***	120.0 ^NS^	337.2
Quercetin	1,777.4 **	2,600.9 ***	201.5 ^NS^	296.5
Tocopherols	4,796.0 ***	365.0 ^NS^	105.0 ^NS^	277.0
Flowering	3,021.0 ***	7,450.0 ***	194.0 ^NS^	94.0
Fruit length	976.1 ^NS^	431.8 ^NS^	20.8 ^NS^	407.8
Fruit weight	507.6 **	194.2 ^NS^	5.4 ^NS^	432.4
Fruit width	2,124.1 ^NS^	352.7 ^NS^	33.9 ^NS^	371.3
Plant height	611.1 ^NS^	853.4 ^NS^	216.1 ^NS^	392.9
Degrees of freedom	2	2	4	60

*C*. *annuum* (n = 4); *C*. *baccatum* (n = 7); *C*. *chinense/C*. *frutescens* (n = 12); S×L: interaction between location and species; NS: not significant;

* significant at *p* ≤ 0.05;

** significant at *p* ≤ 0.01;

*** significant at *p* ≤ 0.001.

Probability (*p*) values were adjusted with a False Discovery Rate (FDR) correction.

**Fig 6:**
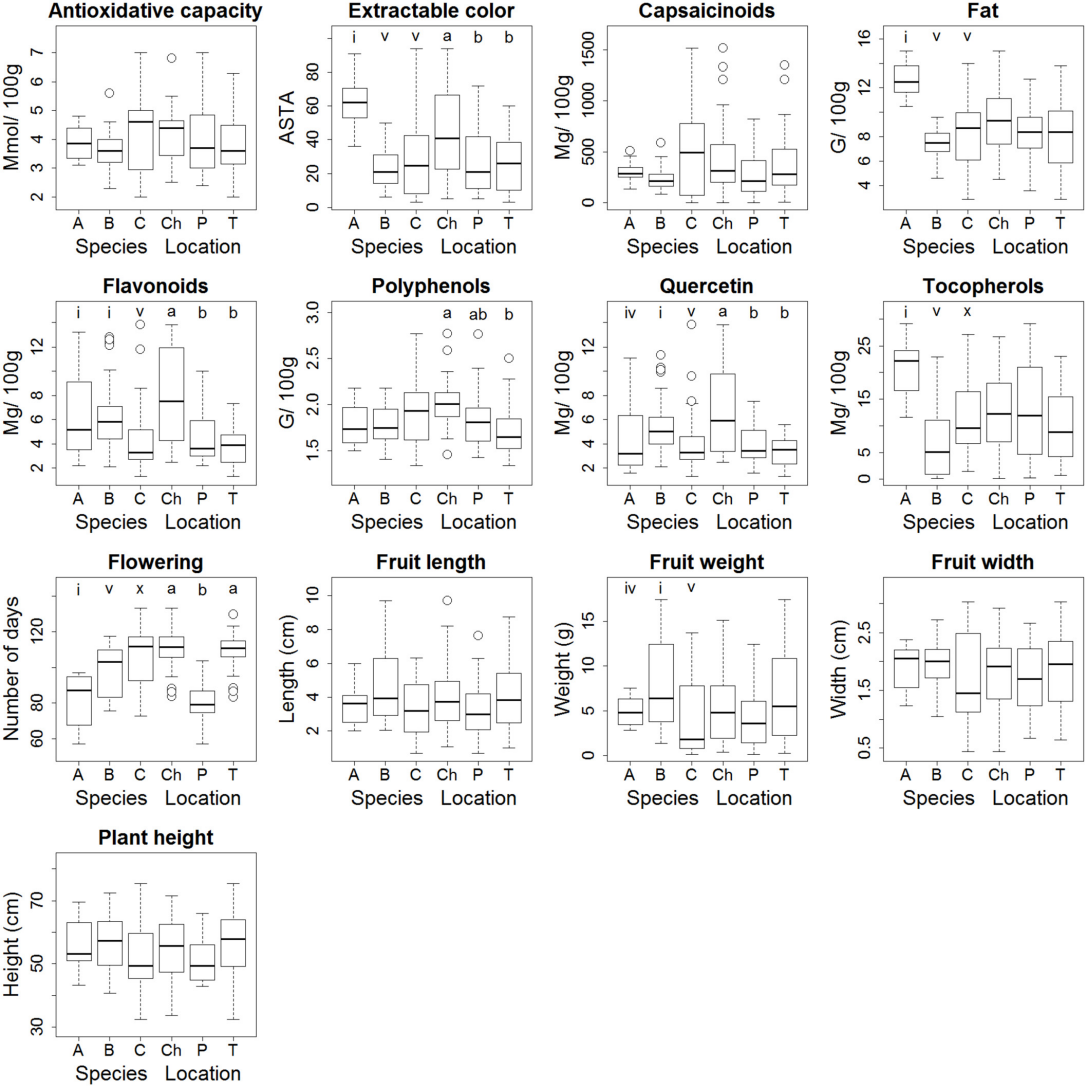
Box plots of trait values per species and per location. Species: A = *C*. *annuum* (n = 4); B = *C*. *baccatum* (n = 7); C = *C*. *chinense/ C*. *frutescens* (n = 12). Locations: Ch = Chiclayo, P = Pucallpa, T = Tambo Grande. Significant differences between species or locations following the two-way ANOVA, are indicated with letters according to post-hoc Turkey’s Honest Significant Difference (HSD) tests with 95% confidence intervals.

The four *C*. *annuum* accessions flowered earlier than the materials of the other species. Their fruits also contained significantly higher fat content and concentrations of tocopherols compared to the other species and expressed an intense red color as indicated by the high values for ASTA extractable color (Fig 6). *Capsicum baccatum* accessions weighed more and contained on average higher flavonoid and quercetin concentrations compared to most *C*. *chinense*–*C*. *frutescens* accessions, but not compared to the *C*. *annuum* accessions. A few *C*. *chinense*–*C*. *frutescens* accessions had exceptionally high flavonoid and quercetin content compared to the other promising accessions (Fig 6).

In the Chiclayo location, the accessions contained higher concentrations of flavonoids and quercetin, and higher values for ASTA extractable color compared to the other sites (Table 5; Fig 6). In Pucallpa, plants flowered earlier in contrast to the other locations. In Chiclayo, higher polyphenol levels were observed compared to Tambo Grande.

Between individual accessions, no differences in environmental variance were detected (F _(22, 264)_ = 1.10; *p* = 0.34). For eight of the 13 traits, the environmental variance did not increase across locations for accessions with high average values; these were antioxidant capacity, levels of capsaicinoids, fat, polyphenols, and tocopherols, flowering time, fruit width, and plant height (Table 6).

**Table 6:** Goodness to fit values of the correlations between environmental variance (*S* ^2^) and mean trait (*m*_*i*_) values across accessions for each attribute.

Attributes	*r* value	*p* value	*p* value corrected
Antioxidant capacity	0.07	0.75	0.75
ASTA extractable color	0.54	< 0.01	0.02
Capsaicinoids	0.42	0.05	0.10
Fat	0.36	0.09	0.14
Flavonoids	0.69	< 0.001	< 0.01
Polyphenols	-0.11	0.62	0.67
Quercetin	0.73	< 0.001	0.001
Tocopherols	0.16	0.45	0.63
Flowering	0.37	0.08	0.14
Fruit length	0.58	< 0.01	0.01
Fruit weight	0.60	< 0.01	0.01
Fruit width	0.14	0.52	0.63
Plant height	0.14	0.53	0.63

Corrected *p* values were adjusted with a False Discovery Rate (FDR) correction.

Accession scores for ASTA extractable color, fat, flavonoids, polyphenols and quercetin were consistent with the first biochemical characterization of these accessions (S6 Table). Antioxidant capacity and capsaicinoid scores were not consistent between the two screenings. We don´t report here on tocopherols because these phytonutrients were only measured in the second screening.

**Fig 7:**
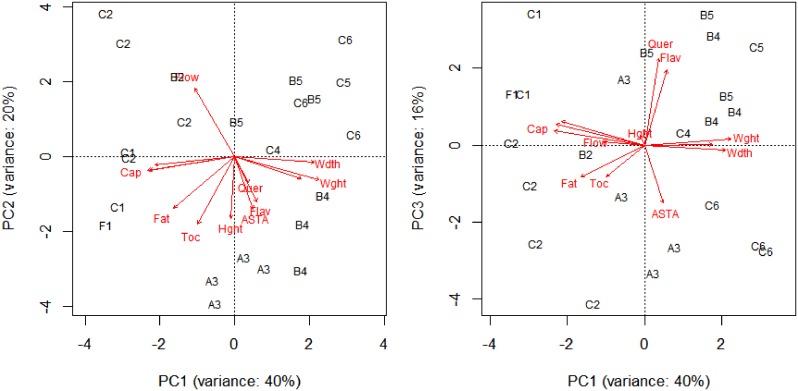
The first three components of a Principal Component Analysis (PCA) of the 23 promising Peruvian accessions. Accessions are ordinated in response to the biochemical and agromorphological variation between them. Accessions are classified per species: A = *C*. *annuum*; B = *C*. *baccatum*; C = *C*. *chinense*; and F = *C*. *frutescens;* and are numbered by the six cluster groups defined by hierarchical clustering. Traits are indicated by abbreviations. ASTA = ASTA extractable color; Cap = capsaicinoids; Fat = fat; Flav = flavonoids; Quer = quercetin; Toc = tocopherols; Hght = Plant height; Flow = 50% plants set flowering; Wdth = fruit width; Wght = fruit weight. Polyphenols and antioxidant capacity score labels overlapped with capsaicinoid score labels and are therefore not shown. Fruit length score labels are not shown because they overlapped with fruit weight and width score labels.

Most variation between promising accessions was seen in capsaicinoid levels and fruit shape and weight as well as the antioxidant capacity and the concentration of polyphenols (Fig 7). The cluster analysis classified the 23 accessions into six groups (Fig 7; Table 7). These are:

1. Small very hot *C*. *chinense*–*C*. *frutescens* peppers with high antioxidant capacity and high levels of polyphenols
2. Piquant *C*. *baccatum*–*C*. *chinense* peppers
3. Semi-piquant *C*. *annuum* peppers with high levels of tocopherols, high fat content, and high ASTA extractable color values
4. Mild *C*. *baccatum*–*C*. *chinense* peppers with big, heavy fruits
5. Mild *C*. *baccatum*–*C*. *chinense* peppers with high concentrations of flavonoids and quercetin
6. Sweet *C*. *chinense* peppers

**Table 7:** Species and mean trait values per group after hierarchical clustering with Euclidian distances and Ward´s grouping method.

Cluster group	1	2	3	4	5	6
Number of accessions	3	5	4	4	4	3
Species	*C*. *chinense*, *C*. *frutescens*	*C*. *baccatum*, *C*. *chinense*	*C*. *annuum*	*C*. *baccatum*, *C*. *chinense*	*C*. *baccatum*, *C*. *chinense*	*C*. *chinense*
Antioxidant capacity (mmol/100g)	6.0	4.6	3.9	3.8	3.2	2.6
ASTA extractable color	17.1	32.5	61.8	29.6	17.0	41.2
Capsaicinoids (mg/100g)	995.0	592.1	307.4	227.9	189.1	33.9
Fat (g/100g)	10.0	9.8	12.6	7.5	6.2	6.2
Flavonoids (mg/100g)	6.5	2.8	6.3	6.4	7.6	3.2
Polyphenols (g/100g)	2.4	2.0	1.8	1.9	1.7	1.5
Quercetin (mg/100g)	6.2	2.7	4.5	5.2	6.6	3.2
Tocopherols (mg/100g)	19.7	9.5	21.1	11.1	1.6	9.0
Flowering (days)	105.8	109.2	82.0	99.4	97.2	104.4
Fruit length (cm)	2.4	2.3	3.6	6.6	3.3	5.0
Fruit weight (g)	1.2	1.3	4.9	12.1	6.0	7.5
Fruit width (cm)	1.1	1.2	1.9	2.2	2.1	2.3
Plant height (cm)	58.5	49.1	55.6	62.6	48.4	50.4

Entrepreneurs expressed specific interest in promising accessions from the cluster groups with low and intermediate pungency, as well as those with high concentrations of health-related biochemical traits including antioxidant capacity, flavonoids, quercetin, and tocopherols.

## Discussion

In this study we have shown how using kinds of phenotypic and geographic diversity as selection criteria is an effective approach to find accessions with a diverse set of features of interest, in a relatively short time span of three years including field collecting and seed regeneration to obtain the necessary diversity for selection. This is particularly useful when screening germplasm from centers of crop diversity because of the high variation found here. In our case, it has allowed in both Peru and Bolivia for the identification of a set of promising *Capsicum* genebank materials for multiple uses including the development of a wide range of fresh and processed food products [31], [45]. Our approach is similar to the development of mini-core collections [46], and differs from more traditional germplasm selection for breeding, which usually focuses on one or two specific traits of interest such as fruit size or pungency in the case of *Capsicum*.

The extensive biochemical analyses allowed us to detect accessions with interesting properties for novel products. A similar approach of using diversity in biochemical properties to select promising accessions has been applied successfully in other *Capsicum* studies [21], [47]. In our study, we were able to take this a step further and evaluate biochemical and agromorphological properties of promising materials in different environments after these accessions were selected from a first biochemical characterization.

Most advances were made with this approach in Peru where we were able to evaluate promising accessions from the *C*. *annuum* and *C*. *baccatum* gene pools in different environments to select elite material. The differences in agromorphological and biochemical performance observed between these accessions are largely the result of the divergent processes of human selection as different species and landraces were domesticated and used in different areas. It is less likely that these differences are a result of natural evolutionary processes. The genetically distinct *C*. *baccatum* and *C*. *chinense* accessions showed much phenotypic similarity, while the *C*. *annuum* accessions distinguished themselves clearly from the other promising materials; the reasons for their particular combination of characteristics require further research.

Although most trait combinations that we observed and the differences between species and accessions can be explained as a result of domestication, some biochemical traits correlate because they functionally relate to each other. Quercetin is a specific flavonoid [30]. The levels of polyphenols and capsaicinoids determine partly the antioxidant capacity of a fruit [48].

The biochemical traits were more influenced by environmental factors than agromorphological attributes, even if we just consider uncorrelated phytochemical traits such as ASTA extractable color, polyphenols, and flavonoids. These results are in line with other studies [49], [50]. Yet, our G *x* E experiment also revealed that for accessions with high antioxidant capacity and high concentrations of fat and tocopherols, and different levels of capsaicinoids, these traits remained stable across environments.

Fat content in fruits of the promising accessions appeared to be consistent over the two biochemical screenings carried out during the selection and evaluation process. Accessions with high fat content are therefore interesting for further evaluation and genetic improvement. On the other hand, although, antioxidant capacity and capsaicinoid levels were stable traits across environments in the G *x* E evaluation, the values of these traits expressed by the promising accessions were not consistent over the two biochemical analyses carried out. The reasons for these contrasting results are not clear and require further evaluation between generations and across different environments for these traits.

Some accessions displayed exceptionally high values of flavonoid and quercetin concentrations and ASTA extractable color in specific locations as demonstrated by their high environmental variance related to high average values across locations (see Table 6). It would be interesting to investigate whether these trait expressions can be linked to the environmental conditions of specific locations. The differences in concentrations of these biochemical compounds and in the level of polyphenols between the peppers in the Chiclayo and Pucallpa locations could possibly be explained by differences in water access. Peppers growing in the desert in Chiclayo were irrigated while in the humid rainforest conditions of Pucallpa they were rain-fed. The content of capsaicinoids is reported to increase when plants suffer from drought, and other phytonutrients may behave similarly [51]. The evaluation sites in both Chiclayo and Tambo Grande were located in the tropical desert but fruits were harvested later in Chiclayo (Table 1; S1 Table). This extra time in Chiclayo may have allowed the plants to develop high concentrations of phytonutrients, as has been reported in other studies [49]. It is difficult to estimate how differences in soil conditions among sites influenced plant performance and biochemical composition of *Capsicum* fruits. More controlled experiments are necessary to understand the interaction between drought, maturation and agromorphological and biochemical properties.

Of all the agronomic traits, flowering time differed mostly between species and among locations. Timing of flowering and fruit set within a season is largely determined by responses to temperature and photoperiod [52]. This explains early flowering in Pucallpa, given the high annual mean temperature compared to the other locations, and the delayed flowering in Huaral, given the low annual mean temperature compared to the other sites, and which led to the retarded fruiting in the latter site (Table 1). Yet, it´s unclear why the *Capsicum annuum* landraces flowered earlier than accessions from the other species in all three sites where accessions produced fruits on time for biochemical evaluation.

Our study focused only on the characterization and evaluation of promising accessions for agronomic and biochemical properties and did not include breeding. It is encouraging, however, that INIA has included all 39 promising *Capsicum* materials identified in this study in their horticultural improvement program. Several promising Peruvian accessions, such as ‘ayuyo’ (*C*. *baccatum*) and ‘ají cerezo’ (*C*. *annuum*) landraces, are already of interest for direct production because they match the interests of national entrepreneurs who participated in project information-sharing workshops. These materials are non-pungent, mild or semi-piquant, have high values for several health-related attributes, and are highly productive compared to other accessions [31]. Other commercially and agronomically important characteristics of the fruits and plants still need to be explored, such as taste profiles and resistance to pests and diseases. Genomic advances in *Capsicum* hold promise to complement selection, evaluation and breeding activities, for example in selecting promising accessions and elite material, which have genes that are related to certain traits of interest [28], [53].

Some promising Bolivian accessions, including several wild *C*. *eximium* and *C*. *baccatum* var. *baccatum* landraces, produced fruits with exceptionally high fat content in specific evaluation sites compared to other Bolivian accessions (Table 4), yet they performed poorly across different environments, which suggests that these landraces only respond well to the local conditions under which they were developed. Further research on landrace–environment interactions and crop improvement is required to develop varieties that perform well in a broader environmental range.

The wild *Capsicum* species of Bolivia deserve special attention. Bolivia is probably the country in the world where humans consume the highest diversity of wild *Capsicum*. Human consumption of *C*. *cardenasii*, the recently identified *C*. *eshbaughii* and *C*. *caballeroi* is exclusive to Bolivia according to current knowledge [6], [42], [54]. *Capsicum eximium* is consumed in Bolivia and Northern Argentina [6]. *Capsicum baccatum* var. *baccatum* and *C*. *chacoense* are commonly harvested and consumed in Bolivia and also in Argentina, Paraguay and Brazil (D.E. Williams, pers. comm.). In many traditional communities throughout Latin America, wild and semi-wild *Capsicum* species are often found growing in close proximity to cultivated peppers, in home gardens or at the edges of cultivated fields, where introgressive hybridization takes place between the cultigen and closely related wild species or sub-species, spontaneously giving rise to novel or intermediate forms which are then selected and propagated by observant farmers in an ongoing process of on-farm crop evolution [55].

Many wild *Capsicum* species are already recognized by plant breeders as useful material for the genetic improvement of cultivated peppers [5], [56]. These wild genetic resources also present an opportunity to domesticate, breed, and cultivate novel pepper varieties with high market potential [41].

As a result of the taxonomic identification of the Bolivian *C*. *baccatum* collection, several accessions of supposedly wild ‘arivivi’ have been revealed to be in fact small-fruited landraces of the cultivated *C*. *baccatum* var. *pendulum*. These landraces are promising candidates for commercial cultivation to reduce the uncontrolled extraction pressure on wild populations of *C*. *baccatum* and *C*. *chacoense* (this latter species is also known as ‘arivivi’). Sustaining ‘ulupica’ (*C*. *caballeroi*, *C*. *cardenasii*, *C*. *eshbaughii*, and *C*. *eximium*) production is a greater challenge because these species have not yet been domesticated. Further work is required to develop commercially and ecologically sustainable extraction and cultivation systems for wild *Capsicum* species.

## Conclusions

With the use of phenotypic and geographic diversity as selection criteria, we were able to identify promising *Capsicum* accessions in Peru and Bolivia for a potentially wide range of high-value products within a short time span of three years. The strengthened genebank collections in the center of cultivated *Capsicum* diversity provided the necessary variation for selection. Biochemical analysis allowed us to identify accessions with promising traits beyond the basic distinction between sweet and hot peppers. These accessions are relevant for novel food, health and other products.

Although we used the same approach for germplasm screening in Bolivia and Peru, the differences in *Capsicum* diversity between the two countries, distinct local environmental conditions, and culture and market opportunities led to different outcomes from the selection process. The promising accessions resulting from this process are the basis for national and local genetic improvement programs, which ideally should be developed in collaboration with entrepreneurs and farmers to ensure that the developed varieties match both market and farmers’ needs.

In Peru, some accessions are already being used for commercial production because of their high productivity and the interest shown by entrepreneurs. These include several sweet, mild and semi-piquant peppers with high concentrations of flavonoids and quercetin or high levels of tocopherols. These accessions produced their highest concentrations of these compounds in an irrigation-controlled system in the tropical desert in Chiclayo. Rain-fed systems in the Amazon provide a good alternative as a low-input system. In Bolivia, the selected cultivated and wild pepper landraces only performed well in specific environments, suggesting that they are strongly adapted to local conditions. Wild *Capsicum* harvesting requires further research for sustainable management.

Our study highlights the limited knowledge about the breeding potential and potentially useful traits present in crop gene pools, even for globally important crops like *Capsicum* peppers. Many opportunities remain to further screen and evaluate genetic resources from *Capsicum*´s center of diversity. Similar opportunities are awaiting for the under-researched genetic resources of other crops in their respective centers of diversity.

## Supporting Information

S1 Dataset Biochemical characterization and passport data of the Bolivian and the Peruvian representative subsets. (CSV)

S2 Dataset Morphological evaluation data of the 23 promising Peruvian accessions at the three repetitions within each of the three locations. (CSV)

S3 Dataset Morphological and biochemical evaluation data of the 23 promising Peruvian accessions at the three locations. (CSV)

S1 Table Environmental descriptors of the Peruvian genotype by environment trials. (DOCX)

S2 Table Environmental descriptors of the Bolivian genotype by environment trials. (DOCX)

S3 Table Comparison of the number of accessions per species reported in this study with two important international *Capsicum* collections. Data were retrieved from the respective genebank information systems at 3 September 2014.
(DOCX)

S4 Table Probability values (*p* values) that biochemical attributes in the set of promising accessions are similar to the representative subset; *t* tests were applied separately for each attribute. (DOCX)

S5 Table One-way ANOVAs with nested design to verify differences among the three repetitions between and within the Peruvian evaluation sites for the agromorphological attributes. (DOCX)

S6 Table Probability values (*p* values) that the order in values for biochemical attributes of the promising materials in the genotype by environment evaluation and the initial biochemical characterization are consistent with each other; *t* tests were applied separately for each attribute. (DOCX)
