# Supplementary material for: Screening Genetic Resources of Capsicum Peppers in Their Primary Center of Diversity in Bolivia and Peru
Source: PLoS One. 2015 Sep 24;10(9):e0134663. doi: 10.1371/journal.pone.0134663 (PMC4581705; doi:10.1371/journal.pone.0134663)
Supplement: S1 Table — (DOCX) [file pone.0134663.s004.docx]

**S1 Table**. Environmental descriptors of the Peruvian genotype by environment trials.

| **Descriptor** | **Chiclayo** | **Piura** | **Pucallpa** | **Huaral** |
| --- | --- | --- | --- | --- |
| Sowing date | 05-05-2012 | 05-05 -2012 | 30-04-2012 | 10-06-2012 |
| Transplanting date | 19-06-2012 | 20-06-2012 | 22-06-2012 | 12-08-2012 |
| harvest date | 08-11-2012 and 17-12-2012 | Last week of October 2012 | First week of December 2012 | 28-01-2013 |
| Fertilizer application | Organic: 200 kg of manure at the start, a second application after 20 days and a third at the start of flowering. | Three times. First: at the start. Second: 11 kg urea, 7 kg di-ammonium potassium, 10 kg potassium sulfate. Third: 6 kg urea, 3 kg di-ammonium phosphate, 20 kg potassium sulphate. | Organic: 150 kg of chicken manure at the start. Each 15 days: abonofol (0.2%) until fruits started to ripen. | In soil preparation, 1 ton of farmyard manure is applied. At 20 days after planting, were applied 120-80-60 using sources of ammonium nitrate, ammonium phosphate and potassium chloride (50% Nitrogen 100 Phosphorus and Potassium 100%). |
| Irrigation method | Gravity irrigation | Drip irrigation | Unirrigated | Gravity irrigation |
| Irrigation Frequency | A total of 800 m^3^ with intervals of 15 days (10 times in total) | A total of 400 m^3^ at irregular intervals depending on the need of water |  | 1900 m^3^ - with intervals of 8 days (24 times in total) |
| Pest and disease management | Integrated pest management | Integrated pest management | Integrated pest management | Integrated pest management |
| Soil parent material (Unconsolidated material and Rock type) | In situ weathered and limestone | Alluvial deposits and unknown rock type | Fluvial deposits and unknown rock type | Aeolian sand, others: Silicates |
| Soil drainage | Moderately drained | Well drained | Moderately drained | Moderately drained |
| Soil depth to groundwater table | 50 – 100 cm | > 150 cm | 50 - 150 cm | 0 - 25 cm |
| Soil salinity | 160 – 240 ppm | <160 ppm | <160 ppm | <160 ppm |
| Soil erosion | Low | Low | Low | Low |
| Soil texture | Clay | Sandy clay | Clay | Fine sandy loam |
