# Supplementary material for: Screening Genetic Resources of Capsicum Peppers in Their Primary Center of Diversity in Bolivia and Peru
Source: PLoS One. 2015 Sep 24;10(9):e0134663. doi: 10.1371/journal.pone.0134663 (PMC4581705; doi:10.1371/journal.pone.0134663)
Supplement: S2 Table — (DOCX) [file pone.0134663.s005.docx]

**S2 Table**. Environmental descriptors of the Bolivian genotype by environment trials.

| **Descriptor** | **Monteagudo** | **Padilla** | **Cochabamba** | **Santa Cruz** |
| --- | --- | --- | --- | --- |
| Sowing date | 15-10-2011 | 15-10-2011 | 10-10-2011 | 10-10-2011 |
| Transplanting date | 30-12-2011 | 19-12-2011 | 11-04-2012 | 28-04-2012 |
| harvest date | 10-07-2012 | 29 -06- 2012 | First 24-08-2012 and last 21-02-2013 | From 01-09- 2012 |
| Fertilizer application | Organic: 4 t/ha, 50% at planting and 50% at the time of hilling Foliar: 3 times: at the beginning of the plant, 2^nd^ before flowering, both with 30-10-10 in doses of 1 l/ha (Clink Nitro); 3rd in bloom with 15-30-15 in doses of 1 l/ha (phosphole). Note: Not inorganic fertilization was done. | Organic: 4 t/ha, 50% at planting and 50% at the time of hilling Foliar: 3 times: at the beginning of the plant, 2^nd^ before flowering, both with 30-10-10 in doses of 1 l/ha (Clink Nitro); 3rd in bloom with 15-30-15 in doses of 1 l/ha (phosphole). Note: Not inorganic fertilization was done. | Bi-VIGORTOP organic foliar fertilizer, 2 times (July and August). | Application of compost 3-4 kg per m^2^ per plant before transplanting and foliar fertilizer (Bigotop). |
| Irrigation method | Unirrigated | Unirrigated | Gravity irrigation | Gravity irrigation |
| Irrigation Frequency | Zero | 4 times. A total of 28.8 m^3^ | Each 10 days, unquantified | Each 10-15 days, unquantified |
| Pest and disease management | Insect Control: 3 times:  1) Systemic insecticide Curacron, doses of 0.5 l/ha, 30 days after transplantation. 2) Contact Insecticide Nurelle, doses of 0.5 l/ha at the beginning of pod formation. 3)  Curacron systemic insecticide, dose of 0.5 l/ha, at the time of physiological maturity of the fruit.  Disease control:  1) Priori (Systemic fungicide), 0.5 l/ha at 30 days after transplantation. 2) Curathane (Systemic fungicide), dose of 1 kg/ha at the beginning of flowering. 3) Fungicide | Insect Control: 3 times: 1) with Nurelle 0.5 l/ha at the beginning of fruit formation. 2) Curacron, 0.5 l/ha, in whole fruits. 3) Nurelle 0.5 l/ha, at physiological maturity.    Disease control:  1) Priori (Systemic fungicide), 0.5 l/ha at 30 days after transplantation 2)  Priori, 05 l/ha, in the maturity of the fruit. | No chemical treatment because of organic farming | 2 applications with Actara, 15 g per 20 liters, 1 time with insecticide Sipertrin, 25 ml per 20 liters. |
| Soil parent material (Unconsolidated material and Rock type) | NA | NA | NA | Aeolian sand |
| Soil drainage | Well drained | Moderately drained | Moderately drained | Well drained |
| Soil depth to groundwater table | 50.1 - 100 cm | 50.1 - 100 cm | NA | NA |
| Soil salinity | <160 ppm | <160 ppm | Fe= 56 ppm (high) Zn= 4.2 ppm (high)  Mn= 76 ppm (high) S-SO4= 6 ppm (half)  Cu= 1.7 ppm (high) B= 8.54 ppm (high) | NA |
| Soil erosion | Intermediate | Low | Low | Intermediate |
| Soil texture | Clay loam | Silt loam | Silty clay loam | Sandy loam |
