# Supplementary material for: Screening Genetic Resources of Capsicum Peppers in Their Primary Center of Diversity in Bolivia and Peru
Source: PLoS One. 2015 Sep 24;10(9):e0134663. doi: 10.1371/journal.pone.0134663 (PMC4581705; doi:10.1371/journal.pone.0134663)
Supplement: S3 Table — Data were retrieved from the respective genebank information systems at 3 September 2014. (DOCX) [file pone.0134663.s006.docx]

### S3 Table. Comparison of the number of accessions per species reported in this study with two important international *Capsicum* collections. Data were retrieved from respective information at 3 September 2014.

| **Accessions** | **Complete AVRDC* collection** | **Peruvian AVRDC accessions** | **Bolivian AVRDC accessions** | **Complete USDA** collection** | **Peruvian USDA accessions** | **Bolivian USDA accessions** |
| --- | --- | --- | --- | --- | --- | --- |
| *C. annuum* | 5,442 | 39 | 9 | 4,028 | 30 | 10 |
| *C. baccatum* | 390 | 100 | 46 | 441 | 98 | 53 |
| *C. cardenasii* | 0 | 0 | 0 | 4 | 0 | 4 |
| *C. chacoense* | 25 | 0 | 6 | 22 | 0 | 10 |
| *C. chinense* | 505 | 151 | 21 | 516 | 130 | 27 |
| *C. eximium* | 4 | 0 | 0 | 10 | 0 | 7 |
| *C. flexuosum* | 0 | 0 | 0 | 7 | 0 | 0 |
| *C. frutescens* | 741 | 14 | 1 | 670 | 46 | 8 |
| *C. galapagoense* | 2 | 0 | 0 | 2 | 0 | 0 |
| *C. lanceolatum* | 1 | 0 | 0 | 1 | 0 | 0 |
| *C. praetermissum* | 9 | 0 | 0 | 0 | 0 | 0 |
| *C. pubescens* | 30 | 5 | 1 | 133 | 20 | 11 |
| *C. rhomboideum* | 0 | 0 | 0 | 2 | 0 | 0 |
| *C. schottianum* | 0 | 0 | 0 | 1 | 0 | 0 |
| *C. tovarii* | 3 | 2 | 0 | 1 | 1 | 0 |
| *Capsicum* spp. | 1,083 | 19 | 4 | 356 | 14 | 38 |
| **Total** | **8,235** | **330** | **88** | **6,194** | **339** | **168** |
| * AVRDC is the acronym of the World Vegetable Center | | | | | | |
| ** USDA is the acronym for the United States Department of Agriculture | | | | | | |
