# Supplementary material for: Screening Genetic Resources of Capsicum Peppers in Their Primary Center of Diversity in Bolivia and Peru
Source: PLoS One. 2015 Sep 24;10(9):e0134663. doi: 10.1371/journal.pone.0134663 (PMC4581705; doi:10.1371/journal.pone.0134663)
Supplement: S4 Table — (DOCX) [file pone.0134663.s007.docx]

### S4 Table. Probability values (*p* values) that biochemical attributes in the set of promising accessions are different from the representative subset; *t* tests were applied separately for each attribute.

|  | Peru |  | Bolivia |  |
| --- | --- | --- | --- | --- |
| Attribute | *p* values | *p* values corrected | *p* values | *p* values corrected |
| Antioxidant capacity | 0.04 | 0.22 | 0.35 | 0.61 |
| ASTA extractable color | 0.89 | 0.94 | 0.15 | 0.58 |
| Capsaicinoids | 0.09 | 0.22 | 0.23 | 0.58 |
| Fat | 0.09 | 0.22 | 0.53 | 0.73 |
| Flavonoids | 0.94 | 0.94 | 0.65 | 0.73 |
| Polyphenols | 0.31 | 0.55 | 0.25 | 0.58 |
| Quercetin | 0.91 | 0.94 | 0.73 | 0.73 |
| Corrected *p* values were adjusted with a False Discovery Rate (FDR) correction. | | | | |
