# Supplementary material for: Screening Genetic Resources of Capsicum Peppers in Their Primary Center of Diversity in Bolivia and Peru
Source: PLoS One. 2015 Sep 24;10(9):e0134663. doi: 10.1371/journal.pone.0134663 (PMC4581705; doi:10.1371/journal.pone.0134663)
Supplement: S5 Table — (DOCX) [file pone.0134663.s008.docx]

**S5 Table**. One-way ANOVAs with nested design to verify differences among the three repetitions between and within the Peruvian evaluation sites for the agromorphological attributes.

| Attribute | Between sites | Within sites | Residuals |
| --- | --- | --- | --- |
| Flowering | 714 ^NS^ | 58,814*** | 1,934 |
| Fruit length | 3,200 ^NS^ | 6,054 ^NS^ | 3,517 |
| Fruit weight | 71 ^NS^ | 4,324 ^NS^ | 3,601 |
| Fruit width | 760 ^NS^ | 4,603 ^NS^ | 3,586 |
| Plant height | 30,331*** | 39,134*** | 2,241 |
| Degrees of freedom | 2 | 6 | 198 |
| Probability (*p*) values were adjusted with a False Discovery Rate (FDR) correction. | | | |

.
