# Supplementary material for: Screening Genetic Resources of Capsicum Peppers in Their Primary Center of Diversity in Bolivia and Peru
Source: PLoS One. 2015 Sep 24;10(9):e0134663. doi: 10.1371/journal.pone.0134663 (PMC4581705; doi:10.1371/journal.pone.0134663)
Supplement: S6 Table — (DOCX) [file pone.0134663.s009.docx]

### S6 Table. Probability values (*p* values) that the order in values for biochemical attributes in the set of promising materials is different from the representative subset; *t* tests were applied separately for each attribute.

| Trait | *p* values | *p* values corrected |
| --- | --- | --- |
| Antioxidant capacity | 0.002 | 0.017 |
| ASTA extractable color | 0.75 | 0.84 |
| Capsaicinoids | 0.01 | 0.04 |
| Fat | 0.23 | 0.32 |
| Flavonoids | 0.10 | 0.18 |
| Polyphenols | 0.84 | 0.84 |
| Quercetin | 0.06 | 0.14 |
| Corrected *p* values were adjusted with a False Discovery Rate (FDR) correction | | |
